# The ‘super acid’ BF_3_H_2_O stabilized by 1,4-dioxane: new preparative aspects and the crystal structure of BF_3_H_2_O·C_4_H_8_O_2_

**DOI:** 10.1107/S2056989019014312

**Published:** 2019-10-31

**Authors:** Peter Barthen, Walter Frank

**Affiliations:** a Institut für Anorganische Chemie und Strukturchemie, Lehrstuhl II: Material- und Strukturforschung, Heinrich-Heine-Universität Düsseldorf, Universitätsstrasse 1, D-40225 Düsseldorf, Germany

**Keywords:** crystal structure, mono­aqua­tri­fluorido­boron, boron trifluoride monohydrate, dioxane solvate, super acid, hydrogen bonding, chain structure

## Abstract

Highly Brønsted-acidic boron trifluoride monohydrate, a widely used ‘super acid-catalyst’, is a colourless fuming liquid that releases BF_3_ at room temperature. Com­pared to the liquid com­ponents, *i.e.* boron trifluoride monohydrate and 1,4-dioxane, their 1:1 adduct, BF_3_H_2_O·C_4_H_8_O_2_, is a solid with pronounced thermal stability (m.p. 401–403 K). The crystal structure of the long-time-stable easy-to-handle and weighable com­pound is reported along with new preparative aspects and the results of ^1^H, ^11^B, ^13^C and ^19^F spectroscopic investigations, particularly documenting its high Brønsted acidity in aceto­nitrile solution. The remarkable stability of solid BF_3_H_2_O·C_4_H_8_O_2_ is attributed to the chain structure established by O—H⋯O hydrogen bonds of exceptional strength {O2⋯H1—O1 [O⋯O = 2.534 (3) Å] and O1—H1⋯O3^i^ [2.539 (3) Å] in the concatenating unit >O2⋯H1—O1—H2⋯O3^i^<}, taking into account the mol­ecular (non-ionic) character of the structural moieties. Indirectly, this structural feature documents the outstanding acidification of the H_2_O mol­ecule bound to BF_3_ and reflects the super acid nature of BF_3_H_2_O. In detail, the *C*_2_^2^(7) zigzag chain system of hydrogen bonding in the title structure is characterized by the double hydrogen-bond donor and double (κ*O*,κ*O*′) hydrogen-bond acceptor functionality of the aqua ligand and dioxane molecule, respectively, the almost equal strength of both hydrogen bonds, the approximatety linear arrangement of the dioxane O atoms and the two neighbouring water O atoms. Furthermore, the approximately planar arrangement of B, F and O atoms in sheets perpendicular to the *c* axis of the ortho­rhom­bic unit cell is a characteristic structural feature.

## Abstract 2

The crystal structure of BF_3_H_2_O·C_4_H_8_O_2_ – the dioxane adduct of the ’super acid’ BF_3_H_2_O – is reported along with new preparative aspects and results of ^1^H, ^11^B and ^13^C and ^19^F spectroscopic investigations. The pronounced thermal stability of the solid adduct (m.p. 128–130 °C) in com­parison to the liquid com­ponents is attributed to the chain structure established by O—H⋯O hydrogen bonds of exceptional strength taking into account the mol­ecular (non-ionic) character of the structural moieties.

## Chemical context   

Solutions of boron trifluoride in water have been under investigation for more than 200 years (Gay-Lussac & Thenard, 1809 ▸; Davy, 1812 ▸; Berzelius, 1824 ▸). Meerwein (1933 ▸) was able to isolate the BF_3_ dihydrate and, after addition of one further equivalent of BF_3_ at low temperature, the BF_3_ monohydrate also. Both hydrates were examined in detail (Klinkenberg & Ketelaar, 1935 ▸; McGrath *et al.*, 1944 ▸; Greenwood & Martin, 1951 ▸; Wamser, 1951 ▸; Pawlenko, 1959 ▸) and while the dihydrate was shown to be distillable without decom­position under reduced pressure, boron trifluoride monohydrate releases BF_3_ above its melting point of 279.2 K. At room temperature, it is a colourless fuming liquid with a density of 1.8 g ml^−1^. To examine the acidity of the monohydrate, reactions with ethers, alcohols and carb­oxy­lic acids *etc*. were performed by Meerwein & Pannwitz (1934 ▸). They obtained BF_3_H_2_O·C_4_H_8_O_2_, which they called the *dioxane salt of boron trifluoride monohydrate*, by adding BF_3_H_2_O to a solution of 1,4-dioxane in petroleum naphta. BF_3_H_2_O·C_4_H_8_O_2_ (**1**) precipitates as needle-shaped crystals which melt at 401–403 K with decom­position (Meerwein & Pannwitz, 1934 ▸). Unexpectedly, the experiment described in §6 resulted in the same product. The primordial idea of this experiment was to prepare an anhydrous solution of HBF_4_ from HBF_4_/H_2_O (1:1 *w*:*w*) by distilling off water as the 1,4-dioxane/water azeotrope with coincident replacement of water by an excess of 1,4-dioxane. The dioxane adduct **1** starts to precipitate after a short period of time if a small amount of water remains in the resulting liquid. The formation of **1** in a ‘HBF_4_ solution’ impressively illustrates how efficently BF_3_ is stabilized by water and dioxane. The reactions and equilibria of HBF_4_-, BF_3_-, H_2_O- and HF-containing systems have been examined in detail (Pawlenko, 1968 ▸; Gascard & Mascherpa, 1973 ▸; Christe *et al.*, 1975 ▸; Mootz & Steffen, 1981*a* ▸; Yeo & Ford, 2006 ▸; Dubey *et al.*, 2007 ▸) and it remains amazing that BF_3_H_2_O, unlike the other boron trihalide/water mixtures, releases the strong Lewis-acid (BF_3_) unhydrolysed. Investigations by Greenwood & Martin (1951 ▸) showed that BF_3_H_2_O is highly ionized in the liquid state and that the Hammett acidity of H[BF_3_OH] is *H_0_* = −11.4. By NMR spectroscopic determination of the thermodynamic acidity function from ^13^C chemical-shift changes of the signals of unsaturated ketones at infinite dilution in the acid under investigation, Farcasui & Ghenciu (1992 ▸) found boron trifluoride monohydrate to be super acidic, with *H_0_* < −14. The applications of this super acid are numerous, *e.g.* as a highly effective catalyst for several Friedel–Craft reactions (Yoneda *et al.*, 1969 ▸; Oyama *et al.*, 1978 ▸; Liu *et al.*, 2003 ▸; Prakash *et al.*, 2016 ▸, and references therein). The long-time-stable and easy-to-handle solid **1** provides the ‘super acid BF_3_H_2_O’ in a safe and efficient way.
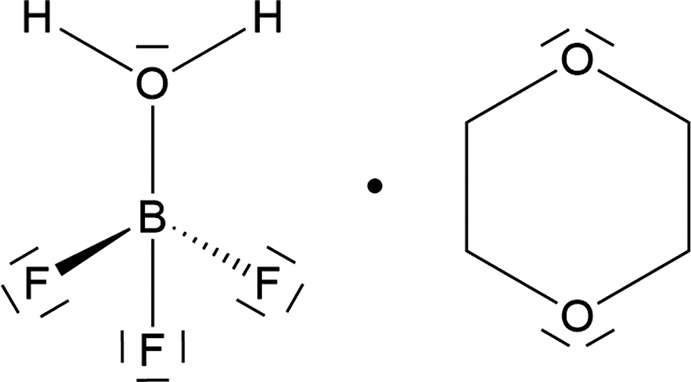


Although Meerwein & Pannwitz (1934 ▸) isolated com­pound **1** (m.p. 401–403 K) and a solid, in which BF_3_H_2_O is stabilized by 1,8-cineole (m.p. 344–346 K) more than 80 years ago, the crystal structures of these com­pounds are still unknown and the reasons for the unexpected high thermal stability, especially of the dioxane adduct, are still unknown. Generally, there are very rare examples of crystal structures with BF_3_H_2_O moieties bound to O-donor mol­ecules. The crystal structure of boron trifluoride monohydrate itself has been reported by Mootz & Steffen (1981*b* ▸), after redetermination of the crystal structure of the dihydrate in the same year (Mootz & Steffen, 1981*c* ▸; Bang & Carpenter, 1964 ▸). Stabilization of the mono- and dihydrate with 18-crown-6 (Bott *et al.*, 1991 ▸; Feinberg *et al.*, 1993 ▸; Simonov *et al.*, 1995 ▸) or of BF_3_H_2_O with di­cyclo­hexane-18-crown-6 (Fonar *et al.*, 1997 ▸) led to three further crystal structures containing the BF_3_H_2_O moiety and, as the most recent example, stabilization with tri­phenyl­phosphane oxide (Chekhlov, 2005 ▸) gave a crystalline 1:2 adduct of BF_3_H_2_O and (C_6_H_5_)_3_PO.

## Structural commentary   

**Figure 1:**
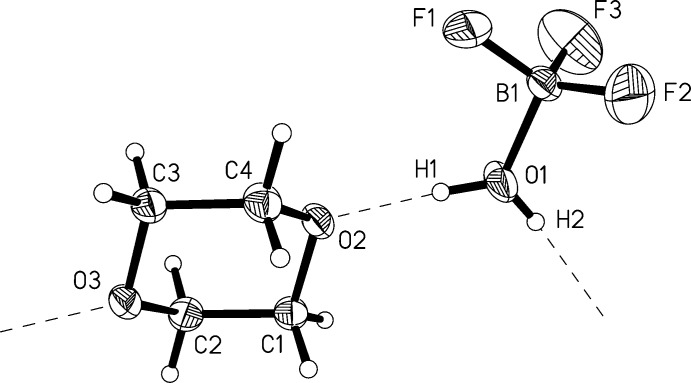
Diagram of the asymmetric unit of the crystal structure of com­pound **1**, displaying the atom-labelling scheme. Anisotropic displacement ellipsoids are drawn at the 40% probability level and the radii of H atoms are chosen arbitrarily. The direction of hydrogen bonding is given by dashed lines.

Compound **1** was found to crystallize in the ortho­rhom­bic space group *Pbca* with eight formula units in the unit cell and all com­ponents in general positions. Fig. 1 ▸ shows the asymmetric unit of the crystal structure, which contains aqua­tri­fluorido­boron and 1,4-dioxane *mol­ecular* moieties. The dioxane moiety is free of any kind of conformational disorder often recognized in the case of saturated six-membered ring species. Bond lengths, angles and torsion angles defining the chair conformation are in excellent agreement with the expectations for a ‘fully ordered’ dioxane mol­ecule, *e.g.* found in the structure of uncom­plexed 1,4-dioxane at 153 K (Buschmann *et al.*, 1986 ▸). Compared to the mean equivalent isotropic displacement parameter (*U*_eq_) of the C and O atoms in the 1,4-dioxane moiety [= 0.0427 (6) Å^2^], the mean *U*_eq_ value of B1, O1 and F1 to F3 in the aqua­tri­fluorido­boron moiety [0.0867 (8) Å^2^] is dramatically higher and correction for libration is needed prior to com­parison with the geometries of BF_3_H_2_O moieties in related com­pounds. In Table 1 ▸, the uncorrected and corrected (Schomaker & Trueblood, 1968 ▸; RG = 0.0241) B—O and B—F bond lengths of **1** are given in com­parison to the bond lengths of BF_3_H_2_O (Mootz & Steffen, 1981*c* ▸) and BF_3_H_2_O·H_2_O (Mootz & Steffen, 1981*b* ▸). After correction, the values of **1** agree well with those of the hydrates and those in almost undistorted BF_4_^−^ as found in Li[BF_4_] at 200 K [1.387 (3)–1.391 (3) Å; Matsumoto *et al.*, 2006 ▸] or in H_5_O_2_[BF_4_] [1.381 (2)–1.399 (2) Å; Mootz & Steffen, 1981*a* ▸]. The bond-valence sum of B1 is as expected taking into account the ‘uncorrected’ nature of the *r*_0_ values used (Brown & Altermatt, 1985 ▸). Inter­estingly, for all com­pounds mentioned in Table 1 ▸, the B—F bond perpendicular to the plane of the aqua ligand (**1**: B1—F3; BF_3_H_2_O: B1—F2; BF_3_H_2_O·H_2_O: B1—F3) is slightly but significantly longer than the other two B—F bonds, probably attributable to a small destabilizing inter­action with the oxygen lone pair. The F—B—O angles in all three com­pounds [**1**: 105.6 (3)–109.8 (3)°; BF_3_H_2_O: 105.9 (4)–108.1 (4)°; BF_3_H_2_O·H_2_O: 106.3 (1)–109.8 (1)°] are smaller than the F—B—F angles [**1**: 109.9 (3)–112.1 (3)°; BF_3_H_2_O: 111.2 (4)–113.0 (4)°; BF_3_H_2_O·H_2_O: 109.8 (1)–114.0 (1)°]. This fits to the observation (Table 1 ▸) that the B—O bond in the BF_3_H_2_O moiety is relatively weaker than the B—F bonds and the planar geometry of BF_3_ is preserved in the aqua com­plex to some extent. Furthermore, for all three com­pounds, the O—B—F angle including the F atom that is approximately in plane with the aqua ligand [**1**: O1—B1—F1 = 105.6 (3)°; BF_3_H_2_O: O—B—F3 = 105.9 (4)°; BF_3_H_2_O·H_2_O: O1—B—F2 = 106.3 (1)°] is significantly smaller than the other O—B—F angles. This observation may be attributed to an attractive F⋯H inter­action within the moiety.

**Figure 2:**
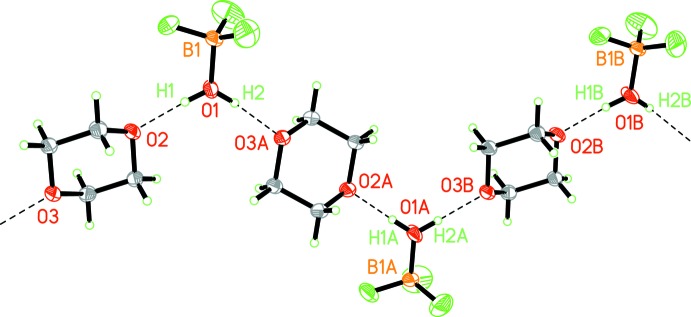
The zigzag chain of hydrogen-bonded moieties in the crystal of **1** [view direction [001]; 30% probability ellipsoids; symmetry codes: (A) −*x* + 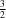, *y* − 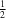, *z*; (B) *x*, *y* + 1, *z*]. Features indicative for the mode of concatenation of the characteristic building blocks by hydrogen bonding are: (i) double hydrogen-bond donor and double (κ*O*,κ*O*′) hydrogen-bond acceptor functionality of the aqua ligand and dioxane moiety, respectively; (ii) almost equal strength of both hydrogen bonds; (iii) an approximatety linear arrangement of the dioxane O atoms and the two neighbouring water O atoms (*e.g.* O1, O3A, O2A and O1A); (iv) an approximately planar arrangement of B1, F1, O1, O2 and O3.

Although both BF_3_H_2_O and 1,4-dioxane are liquids at room temperature, adduct **1** is a solid with a remarkably high melting point (401–403 K), mainly resulting from the concatenation of the mol­ecular com­ponents *via* O—H⋯O hydrogen bonding, as shown in Fig. 2 ▸. The high stability might be correlated to the exceptional strength of both O2⋯H1—O1 [O⋯O = 2.534 (3) Å] and O1—H1⋯O3^i^ [2.539 (3) Å] in the concatenating >O2⋯H1—O1—H2⋯O3^i^< unit. Indirectly, this structural feature documents the outstanding acidification of the H_2_O mol­ecule bound to BF_3_ and reflects the super acid nature of BF_3_H_2_O. Further details of the hydrogen bonding are given in Table 2 ▸. To the best of our knowledge, there is no example of a water ligand bonded to a nonmetal or a metal with the ligand engaged in a hydrogen bond of similar strength (O⋯O < 2.60 Å) to an O atom of a dioxane mol­ecule. In the adduct 18-crown-6·BF_3_H_2_O (m.p. 345 K), mentioned in §1, the aqua ligand is hydrogen bonded to two O-donor atoms and the O⋯O distances are 2.76 and 2.80 Å (Feinberg *et al.*, 1993 ▸). In the structure of BF_3_H_2_O·H_2_O, the nonligating water mol­ecule plays a similar role as bridging species as the dioxane mol­ecule in **1**. The O⋯O distances in the characteristic ⋯H—O—H⋯O(H_2_)⋯H—O—H⋯ unit are 2.631 and 2.643 Å (Mootz & Steffen, 1981*c* ▸), *i.e.* as com­pared to the very strong Brønsted acids fluoro­sulfuric acid [O⋯O = 2.643 (1) Å] or tri­fluoro­methane­sulfonic acid [O⋯O = 2.640 (4) Å] (Bartmann & Mootz, 1990 ▸), for example, the hydrogen bonding is of the same strength in the dihydrate and much stronger in the adduct **1**.

## Supra­molecular features   

**Figure 3:**
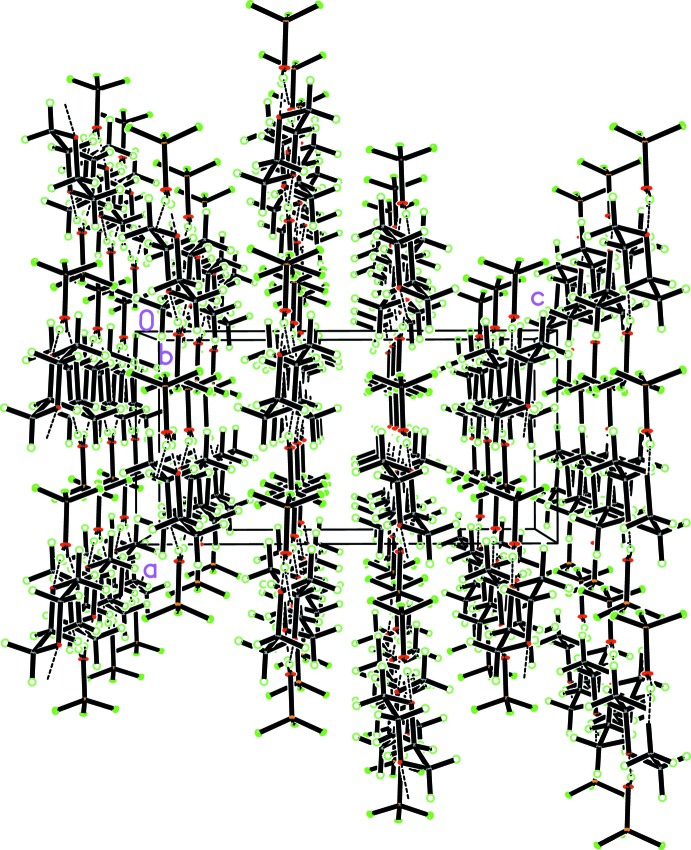
Packing diagram of **1** (view direction [010]) documenting the arrangement of the zigzag chains to flat sheets perpendicular to the *c* axis. Inspection of the inter­molecular distances gives no evidence for inter­actions stronger than van der Waals forces between the chains.

As mentioned before, in the solid of **1** the aqua ligand of the BF_3_H_2_O moiety acts as a hydrogen-bond donor in two directions, establishing a 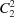
(7) graph set (Etter, 1990 ▸) (Fig. 2 ▸). The propagation vector of the zigzag chain is parallel to the *b* axis of the unit cell. Note the almost equal strength of both hydrogen bonds. Fig. 3 ▸ shows the arrangement of the chains in the solid due to van der Waals inter­actions.

## Database survey   

A search of the Cambridge Structural Database (CSD; Version 5.40, November 2018 update; Groom *et al.*, 2016 ▸) for the BF_3_H_2_O moiety yielded six structures: the crown ether adducts 18-crown-6 mono­aqua­tri­fluorido­boron toluene semisolvate (CSD refcode SIXFOU; Bott *et al.* 1991 ▸), 18-crown-6 bis­(mono­aqua­tri­fluorido­boron) dihydrate (LEKYIJ; Feinberg *et al.* 1993 ▸, Simonov *et al.*, 1995 ▸) and di­cyclo­hexano-18-crown-6 bis­(mono­aqua­tri­fluorido­boron) (NIYGAD; Fonar *et al.*, 1997 ▸); the phosphane oxide adduct mono­aqua­tri­fluorido­boron bis­(tri­phenyl­phosphane oxide) (XATWAR; Chekhlov, 2005 ▸); two transition-metal coordination com­pounds [CIGVUJ10 (Van Rijn *et al.*, 1987 ▸) and UKAJIA (Orain *et al.*, 2010 ▸)], containing cocrystallized mono­aqua­tri­fluorido­boron moieties. As mentioned above, in addition to these reports on com­pounds having organic com­ponents, there is the report of Mootz & Steffen (1981*b* ▸) on the inorganic parent com­pound BF_3_H_2_O and there are two reports on the dihydrate BF_3_H_2_O·H_2_O (Mootz & Steffen, 1981*c* ▸; Bang & Carpenter, 1964 ▸).

## NMR spectroscopy   

NMR studies of BF_3_H_2_O·C_4_H_8_O_2_ have not been published so far. Ford & Richards (1956 ▸) have shown by low-temperature NMR investigations that, in the solid state, BF_3_H_2_O and BF_3_H_2_O·H_2_O are not ionized. Diehl (1958 ▸) reported the ^19^F NMR spectra of BF_3_H_2_O in aqueous solution. He observed separate broad resonances which he attributed to HBF_3_OH, HBF_4_, HBF_2_(OH)_2_ and HBF(OH)_3_ in concentrated solutions at 243 K with coalescence of the peaks at higher temperatures. Gillespie & Hartman (1967 ▸) have shown by low-temperature (193 K) ^1^H and ^19^F NMR spetroscopy that BF_3_H_2_O is formed in dilute solutions in acetone containing both water and BF_3_. They found two major peaks in the ^19^F NMR spectrum and assigned the low-field peak (−146.05 ppm) to the 1:1 com­plex of BF_3_ with acetone and the high-field peak (−146.59 ppm) to BF_3_H_2_O in acetone. The corresponding ^1^H NMR signals were detected by Gillespie & Hartmann at 12.42 ppm as multipletts. In our experiments, in the presence of CD_3_CN *and* 1,4-dioxane and at a significantly higher temperature (297 K), the protons were detected as a broad singlet at 9.41 ppm. Gottlieb *et al.* (1997 ▸) indicated that the influence of temperature on the NMR shift overcom­pensates the influence of the solvent if the basicity of the solvents is similar. Apart from this effect, the high acidity of the oxygen-bonded ^1^H nuclei in the title com­pound is depicted by a shift of more than 7 ppm to higher frequencies (H_2_O in CD_3_CN: *s*, 2.13 ppm; Fulmer *et al.* 2010 ▸). The chemical shifts of the NMR signals belonging to 1,4-dioxane are close to those of the uncom­plexed com­pound (C_4_H_8_O_2_ in CD_3_CN: ^1^H: *s*, 3.60 ppm; ^13^C: 68.5 ppm; Fulmer *et al.*, 2010 ▸). Due to the com­parable donor numbers (Gutmann, 1976 ▸) of aceto­nitrile (NMR solvent) and 1,4-dioxane, it can be concluded that the acidity of BF_3_H_2_O is not critically reduced by 1,4-dioxane with respect to its application as a super acid-catalyst.

The NMR sample was investigated in a 5 mm precision glass NMR tube (Wilmad 507) at 297 K in the deuterium-locked mode on a Bruker Avance III 400 MHz spectrometer operating at 400.17, 376.54, 128.23 or 100.62 MHz for ^1^H, ^19^F, ^11^B and ^13^C nuclei, respectively. The ^1^H NMR and ^13^C chemical shifts were referenced with respect to tetra­methyl­silane yielding the chemical shift for CD_3_CN (contains CD_2_ **H**CN) as 1.96 ppm and CD_3_ **C**N as 118.7 ppm. The ^19^F chemical shifts were referenced with respect to CFCl_3_ (0 ppm) as external standard. The ^11^B chemical shifts were referenced with respect to BF_3_·(C_2_H_5_)_2_O (0 ppm) as external standard. 68 mg of ground crystals were dissolved in 0.5 ml CD_3_CN to prepare the NMR sample: ^1^H NMR: 3.71 (*s*, 8H, C_4_ **H**_8_O_2_), 9.41 (*s*, 2H, **H**_2_O). ^19^F NMR: −148.10 (*s*, ^11^B**F**_3_), −148.04 (*s*, ^10^B**F**_3_). ^11^B NMR: −0.1 (*s*, ^11^ **B**F_3_). ^13^C NMR: 68.0 [*t*, ^1^ *J*_(C,H)_ = 189 Hz, **C**_4_H_8_O_2_].

## Synthesis and crystallization   

All preparations and sample manipulations were carried out in tetra­fluoro­ethyl­ene hexa­fluoro­propyl­ene block copolymer (FEP) vessels. Tetra­fluoro­boric acid solution (50 wt% in water; Fluka Chemicals) was probed for its content of [BF_3_OH]^−^ by ^19^F NMR spectroscopy. Depending on the qu­antity of these anions, hydro­fluoric acid (48 wt% in water, Sigma–Aldrich) was added. In a typical experiment, to 131.4 g (1.24 mol) of HBF_4_/H_2_O, 4.53 g (0.11 mol) HF/H_2_O was added at 273 K. The mixture was stirred for 15 min, before 430 g of 1,4-dioxane was added at the same temperature. Subsequently, the reaction mixture was heated and the 1,4-dioxane–water azeotrope was distilled off under normal pressure until the boiling point (361 K) began to change. 368 g of azeotrope was removed by the distillation and the residue was a pale-brown solution. This solution was stored in a sealed FEP flask under an atmosphere of dry argon (Argon 5.0). After 1 h, the formation of colourless crystals of **1** started and was allowed to continue for 9 d. The crystals were isolated under an argon atmosphere and washed with hexa­ne/1,4-dioxane (10:1 *v*/*v*) three times using Schlenk techniques. 40.7 g (0.23 mol) were collected after drying the almost hexa­gonal colourless crystals in an argon stream (40 min). Compound **1** is stable at room temperature and shows a poor solubility in 1,4-dioxane, but a good solubility in aceto­nitrile.

An elemental analysis was performed with a HEKATECH EA 3000 elemental analyser using *Callidus 2E3* software. 1.7 mg of freshly ground crystals were used and a modifier was added to suppress the influence of the high fluorine content. Analysis calculated (%) for C_4_H_10_BF_3_O_3_: 27.62 C, 5.80 H; found: 27.84 C, 5.87 H.

## Refinement   

Crystal data, data collection and structure refinement details are summarized in Table 3 ▸. The positions of all H atoms were identified *via* subsequent Δ*F* syntheses. In the refinement, a riding model was applied, using idealized C—H bond lengths, as well as H—C—H and C—C—H angles. The *U*_iso_ values were set at 1.2*U*_eq_(C) for methyl­ene H atoms. For the H atoms of the aqua ligand, positional parameters and *U*_iso_ values were refined.

## Supplementary Material

Crystal structure: contains datablock(s) global, I. DOI: 10.1107/S2056989019014312/eb2021sup1.cif

Structure factors: contains datablock(s) I. DOI: 10.1107/S2056989019014312/eb2021Isup2.hkl

CCDC references: 1960541, 1960541

Additional supporting information:  crystallographic information; 3D view; checkCIF report

## Figures and Tables

**Table 1:** Selected bond lengths (Å) Values for BF_3_H_2_O·C_4_H_8_O_2_, BF_3_H_2_O (Mootz & Steffen, 1981*b* ▸) and BF_3_H_2_O·H_2_O (Mootz & Steffen, 1981*c* ▸) in the left, middle and right columns, respectively; in square brackets are the corresponding bond valences and the valence sums calculated using the Brown formalism {*r*_0_[B—O(F)] = 1.371 (1.281), B = 0.37; Brown & Altermatt, 1985 ▸}; in braces are the values corrected for libration (Schomaker & Trueblood, 1968 ▸).

B1—O1	1.473 (4) [0.76] {1.528 (4) [0.65]}	1.532 (6) [0.64]	1.512 (2) [0.68]
B1—F1	1.361 (4) [0.81] {1.409 (4) [0.71]}	1.383 (5) [0.76]	1.377 (2) [0.77]
B1—F2	1.332 (4) [0.87] {1.396 (4) [0.73]}	1.399 (5) [0.73]	1.382 (2) [0.76]
B1—F3	1.333 (4) [0.87] {1.410 (4) [0.71]}	1.382 (5) [0.76]	1.390 (2) [0.74]
			
Σs(B–O,F)	[3.31] {[2.80]}	[2.89]	[2.96]

**Table 2:** Hydrogen-bond geometry (Å, °)

*D*—H⋯*A*	*D*—H	H⋯*A*	*D*⋯*A*	*D*—H⋯*A*
O1—H1⋯O2	0.82 (5)	1.72 (5)	2.534 (3)	175 (5)
O1—H2⋯O3^i^	0.82 (5)	1.72 (5)	2.539 (3)	170 (5)

Symmetry code: (i) 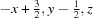.

**Table 3:** Experimental details

Crystal data	
Chemical formula	H_2_BF_3_O·C_4_H_8_O_2_
*M*_r_	173.93
Crystal system, space group	Orthorhombic, *P* *b* *c* *a*
Temperature (K)	223
*a*, *b*, *c* (Å)	7.6835 (5), 12.929 (1), 15.2326 (13)
*V* (Å^3^)	1513.2 (2)
*Z*	8
Radiation type	Mo *K*α
μ (mm^−1^)	0.16
Crystal size (mm)	0.69 × 0.48 × 0.42

Data collection	
Diffractometer	Stoe IPDS
Absorption correction	Multi-scan (Blessing, 1989 ▸)
*T*_min_, *T*_max_	0.673, 0.920
No. of measured, independent and observed [*I* > 2σ(*I*)] reflections	19913, 1481, 932
*R*_int_	0.085
(sin θ/λ)_max_ (Å^−1^)	0.617

Refinement	
*R*[*F* ^2^ > 2σ(*F* ^2^)], *wR*(*F* ^2^), *S*	0.067, 0.138, 1.38
No. of reflections	1481
No. of parameters	108
H-atom treatment	H atoms treated by a mixture of independent and constrained refinement
Δρ_max_, Δρ_min_ (e Å^−3^)	0.60, −0.42

Computer programs: *X-AREA* (Stoe & Cie, 2009 ▸), *SHELXS* (Sheldrick, 2008 ▸), *SHELXL2014* (Sheldrick, 2015 ▸), *SHELXTL* (Sheldrick, 2008 ▸) and *publCIF* (Westrip, 2010 ▸).

## supplementary crystallographic information

### Crystal data

**Table:**

H_2_BF_3_O·C_4_H_8_O_2_	*D*_x_ = 1.527 Mg m^−^^3^
*M**_r_* = 173.93	Mo *K*α radiation, λ = 0.71073 Å
Orthorhombic, *P**b**c**a*	Cell parameters from 7579 reflections
*a* = 7.6835 (5) Å	θ = 2.7–25.9°
*b* = 12.929 (1) Å	µ = 0.16 mm^−^^1^
*c* = 15.2326 (13) Å	*T* = 223 K
*V* = 1513.2 (2) Å^3^	Prisms, colourless
*Z* = 8	0.69 × 0.48 × 0.42 mm
*F*(000) = 720	

### Data collection

**Table:**

Stoe IPDS diffractometer	932 reflections with *I* > 2σ(*I*)
Radiation source: sealed tube	*R*_int_ = 0.085
φ–scan	θ_max_ = 26.0°, θ_min_ = 2.7°
Absorption correction: multi-scan (Blessing, 1989)	*h* = −9→9
*T*_min_ = 0.673, *T*_max_ = 0.920	*k* = −15→15
19913 measured reflections	*l* = −18→18
1481 independent reflections	

### Refinement

**Table:**

Refinement on *F*^2^	Primary atom site location: structure-invariant direct methods
Least-squares matrix: full	Secondary atom site location: difference Fourier map
*R*[*F*^2^ > 2σ(*F*^2^)] = 0.067	Hydrogen site location: difference Fourier map
*wR*(*F*^2^) = 0.138	H atoms treated by a mixture of independent and constrained refinement
*S* = 1.38	*w* = 1/[σ^2^(*F*_o_^2^) + 1.3744*P*] where *P* = (*F*_o_^2^ + 2*F*_c_^2^)/3
1481 reflections	(Δ/σ)_max_ < 0.001
108 parameters	Δρ_max_ = 0.60 e Å^−^^3^
0 restraints	Δρ_min_ = −0.42 e Å^−^^3^

### Special details

**Table:**

Geometry. All esds (except the esd in the dihedral angle between two l.s. planes) are estimated using the full covariance matrix. The cell esds are taken into account individually in the estimation of esds in distances, angles and torsion angles; correlations between esds in cell parameters are only used when they are defined by crystal symmetry. An approximate (isotropic) treatment of cell esds is used for estimating esds involving l.s. planes.

### Fractional atomic coordinates and isotropic or equivalent isotropic displacement parameters (Å^2^)

**Table:**

	*x*	*y*	*z*	*U*_iso_*/*U*_eq_	
F1	0.2228 (2)	0.10315 (15)	0.11881 (16)	0.0760 (7)	
F2	0.2173 (4)	−0.0515 (2)	0.05362 (17)	0.1020 (9)	
F3	0.2125 (4)	−0.0419 (2)	0.19661 (18)	0.1177 (11)	
O1	0.4628 (3)	0.0027 (2)	0.1262 (3)	0.0968 (14)	
H1	0.516 (6)	0.057 (4)	0.133 (3)	0.108 (17)*	
H2	0.515 (6)	−0.053 (4)	0.128 (3)	0.109 (17)*	
O2	0.6367 (2)	0.16850 (14)	0.13995 (14)	0.0435 (5)	
O3	0.8588 (2)	0.33805 (14)	0.11459 (14)	0.0425 (5)	
C1	0.8229 (3)	0.1569 (2)	0.1462 (2)	0.0409 (7)	
H11	0.8705	0.1372	0.0889	0.049*	
H12	0.8511	0.1021	0.1883	0.049*	
C2	0.9021 (4)	0.2567 (2)	0.1756 (2)	0.0435 (8)	
H21	0.8585	0.2745	0.2341	0.052*	
H22	1.0288	0.2493	0.1792	0.052*	
C3	0.6730 (3)	0.3498 (2)	0.1084 (2)	0.0410 (7)	
H31	0.6449	0.4046	0.0663	0.049*	
H32	0.6256	0.3696	0.1657	0.049*	
C4	0.5934 (4)	0.2498 (2)	0.0791 (2)	0.0448 (8)	
H41	0.4666	0.2573	0.0756	0.054*	
H42	0.6366	0.2319	0.0205	0.054*	
B1	0.2712 (4)	0.0021 (3)	0.1232 (3)	0.0412 (8)	

### Atomic displacement parameters (Å^2^)

**Table:**

	*U*^11^	*U*^22^	*U*^33^	*U*^12^	*U*^13^	*U*^23^
F1	0.0439 (11)	0.0559 (12)	0.128 (2)	0.0169 (9)	0.0004 (13)	0.0007 (12)
F2	0.0930 (18)	0.113 (2)	0.1005 (19)	−0.0035 (16)	−0.0243 (16)	−0.0500 (16)
F3	0.118 (2)	0.141 (2)	0.0942 (19)	−0.035 (2)	0.0120 (17)	0.0528 (18)
O1	0.0264 (12)	0.0294 (13)	0.234 (4)	0.0014 (11)	−0.008 (2)	−0.0130 (18)
O2	0.0295 (10)	0.0357 (10)	0.0652 (14)	−0.0044 (8)	−0.0025 (9)	0.0052 (10)
O3	0.0292 (10)	0.0355 (10)	0.0629 (14)	−0.0033 (8)	0.0010 (10)	0.0012 (10)
C1	0.0308 (16)	0.0374 (15)	0.0544 (18)	0.0027 (12)	−0.0045 (13)	0.0001 (14)
C2	0.0314 (14)	0.0430 (17)	0.056 (2)	0.0027 (13)	−0.0078 (14)	−0.0035 (14)
C3	0.0318 (15)	0.0367 (15)	0.0544 (19)	0.0014 (12)	−0.0022 (13)	0.0054 (14)
C4	0.0348 (15)	0.0442 (17)	0.055 (2)	−0.0003 (13)	−0.0100 (15)	0.0025 (14)
B1	0.0299 (16)	0.0422 (18)	0.052 (2)	−0.0037 (15)	−0.0039 (18)	0.0006 (15)

### Geometric parameters (Å, º)

**Table:**

F1—B1	1.361 (4)	C1—C2	1.495 (4)
F2—B1	1.332 (4)	C1—H11	0.9800
F3—B1	1.333 (4)	C1—H12	0.9800
O1—B1	1.473 (4)	C2—H21	0.9800
O1—H1	0.82 (5)	C2—H22	0.9800
O1—H2	0.82 (5)	C3—C4	1.499 (4)
O2—C4	1.440 (3)	C3—H31	0.9800
O2—C1	1.442 (3)	C3—H32	0.9800
O3—C3	1.439 (3)	C4—H41	0.9800
O3—C2	1.442 (3)	C4—H42	0.9800
			
B1—O1—H1	121 (3)	O3—C3—H31	109.8
B1—O1—H2	119 (3)	C4—C3—H31	109.8
H1—O1—H2	120 (4)	O3—C3—H32	109.8
C4—O2—C1	110.4 (2)	C4—C3—H32	109.8
C3—O3—C2	110.4 (2)	H31—C3—H32	108.2
O2—C1—C2	109.5 (2)	O2—C4—C3	110.1 (2)
O2—C1—H11	109.8	O2—C4—H41	109.6
C2—C1—H11	109.8	C3—C4—H41	109.6
O2—C1—H12	109.8	O2—C4—H42	109.6
C2—C1—H12	109.8	C3—C4—H42	109.6
H11—C1—H12	108.2	H41—C4—H42	108.2
O3—C2—C1	110.1 (2)	F3—B1—F2	109.9 (3)
O3—C2—H21	109.6	F3—B1—F1	111.0 (3)
C1—C2—H21	109.6	F2—B1—F1	112.1 (3)
O3—C2—H22	109.6	F3—B1—O1	108.3 (3)
C1—C2—H22	109.6	F2—B1—O1	109.8 (3)
H21—C2—H22	108.2	F1—B1—O1	105.6 (3)
O3—C3—C4	109.5 (2)		
			
C4—O2—C1—C2	58.7 (3)	C2—O3—C3—C4	−58.7 (3)
C3—O3—C2—C1	59.2 (3)	C1—O2—C4—C3	−58.9 (3)
O2—C1—C2—O3	−58.6 (3)	O3—C3—C4—O2	58.5 (3)

### Hydrogen-bond geometry (Å, º)

**Table:**

*D*—H···*A*	*D*—H	H···*A*	*D*···*A*	*D*—H···*A*
O1—H1···O2	0.82 (5)	1.72 (5)	2.534 (3)	175 (5)
O1—H2···O3^i^	0.82 (5)	1.72 (5)	2.539 (3)	170 (5)

Symmetry code: (i) −*x*+3/2, *y*−1/2, *z*.
